# Yeast protein modulates metabolites derived from the human gut microbiota of older male adults *ex vivo* to strengthen gut barrier function and reduce inflammation

**DOI:** 10.3389/fmicb.2025.1697734

**Published:** 2026-01-06

**Authors:** Pieter Van den Abbeele, Lam Dai Vu, Jonas Poppe, Ingmar A. J. van Hengel, Aurélien Baudot, Yan Zhang, Zhixian Chen, Jun Yan

**Affiliations:** 1 Cryptobiotix SA, Ghent, Belgium; 2 The Hubei Provincial Key Laboratory of Yeast Function, Angel Yeast Co. Ltd., Yichang, China; 3 National Key Laboratory of Agricultural Microbiology, Angel Yeast Co. Ltd., Yichang, China

**Keywords:** healthy aging, systemic intestinal fermentation research (SIFR), transepithelial electrical resistance (TEER), short-chain fatty acid (SCFA), branched-chain fatty acids (bCFA), community modulation score (CMS), alternative proteins

## Abstract

**Introduction:**

The rising global demand for protein is accelerating interest in sustainable alternatives with health benefits. While glycans are well-known for supporting gut health, the role of dietary proteins in promoting healthy aging via microbiome modulation is less understood. Yeast protein (YP) represents a sustainable, non-animal, hypoallergenic option.

**Methods:**

Using the clinically predictive *ex vivo* SIFR^®^ technology (Systemic Intestinal Fermentation Research), we examined how YP influences the microbiome of older human adults (50–65 years, *n* = 6), comparing its effects to whey protein isolate (WPI) and soy protein isolate (SPI).

**Results:**

At a dose equivalent to 40 g/day, all protein sources supported gut barrier integrity and reduced inflammation, reflected by decreased pro-inflammatory markers and increased IL-10. These benefits were linked to higher short-chain fatty acid (SCFA) production, mainly from Bacillota and Bacteroidota, including microbial markers associated with healthy aging. YP and SPI specifically restored butyrate-producing microbes and increased microbial diversity, which is linked to longevity. Untargeted metabolomics revealed numerous beneficial amino acid-derived metabolites, including indoles and polyamines, known to act through gut-organ axes to extend health span. Despite similar overall profiles, product-specific differences emerged: YP most strongly reinforced barrier integrity, produced the lowest gas levels (suggesting superior tolerability), and yielded the lowest trimethylamine *N*-oxide, a compound linked to increased mortality in older adults.

**Discussion:**

Collectively, these findings highlight the potential of YP as a sustainable protein source that modulates the microbiome and metabolome, reduces inflammation, and reinforces gut barrier function, which are key mechanisms for preserving health span and mitigating age-related decline.

## Graphical Abstract

**Figure:**
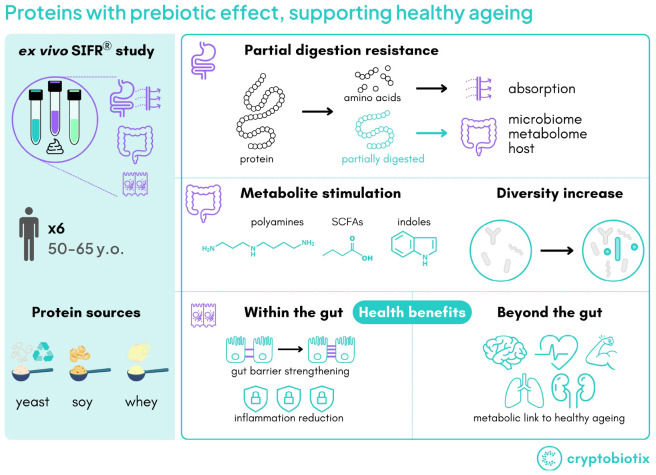
Yeast, soy and whey proteins exerted prebiotic effects *ex vivo*, strengthening the gut barrier and reducing inflammation, highlighting their potential to support healthy ageing.

## 1 Introduction

The human gut microbiome is a complex ecosystem consisting of trillions of microorganisms that altogether impact health (Hou et al., 2022). A well-established function of this microbiome is the fermentation of dietary or host-derived glycans into short-chain fatty acids (SCFA) (Flint et al., 2012), primarily acetate, propionate and butyrate that each have well-established benefits (Rivière et al., 2016). Especially, the benefits of dietary glycans have been extensively studied across different age group (Koropatkin et al., 2012; La Rosa et al., 2022), including the older population (Ale and Binetti, 2021). In contrast, the microbiome-modulating potential of dietary proteins remains underexplored, with emerging evidence suggesting that dietary proteins can also impact the gut microbiome (Bartlett and Kleiner, 2022). While a key role of proteins is to contribute to the maintenance of tissue protein upon digestion by proteases along the small intestine (Bröer, 2023), protein fractions that escape this digestion process reach the colon. Here, they can indeed be fermented by proteolytic gut microbes (Davila et al., 2013; Wang et al., 2019). Besides carbohydrates, various amino acids can serve as substrates for microbial SCFA production (Louis and Flint, 2017). Fermenting branched-chain amino acids (valine, leucine, isoleucine) also produces branched-chain fatty acids (bCFAs), unlike carbohydrate fermentation. Other notable metabolites derived from amino acids include polyamines (from arginine and lysine) (Tofalo et al., 2019) and various indoles (from histidine) (Huang et al., 2024) which has garnered growing scientific interest due to their anti-inflammatory effects as well as impact on systemic health via different gut-organ axes.

A growing number of studies has highlighted the health benefits of proteins, either alone or in combination with other dietary supplements, for the older population (Camargo et al., 2020; Chapman et al., 2021). These benefits are linked to the prevention and management of geriatric syndromes, including impaired gut health (Jones et al., 2024) and metabolic health (Beaudry and Devries, 2019), the loss of muscle mass (sarcopenia) (Ni Lochlainn et al., 2018, 2024; Zhou et al., 2024) and declining neural and cognitive function (Coelho-Júnior et al., 2021; Ni Lochlainn et al., 2024). Additionally, dietary interventions have shown potential in mitigating inflammaging, a chronic, low-grade inflammatory condition linked to persistent activation of the innate immune system, which accelerates cellular senescence (Jukic Peladic et al., 2021; Warman et al., 2022). Importantly, the gut microbiota has been shown to play a crucial role in mediating these positive effects (Ragonnaud and Biragyn, 2021). However, these studies have primarily focused on a limited number of common protein sources. Further, comprehensive multiomics approaches and comparative analyses exploring the impact of diverse protein sources on the human microbiota remain limited (Portune et al., 2016).

Different sources of dietary protein (e.g., beans, vegetables, dairy, seafood or meat) have distinct amino acid profiles, thus likely exerting source-dependent microbiome modulation (Madsen et al., 2017). Yeast protein (YP), derived from *Saccharomyces cerevisiae*, is a protein source of particular interest, containing up to 70% protein, with essential amino acid scores nearly matching the FAO/WHO ideal model (Jach et al., 2022; Xia et al., 2023). YP has high nutrition value, similar to whey protein and superior Digestible Indispensable Amino Acid Score (DIAAS) and Protein Digestibility Corrected Amino Acid Score (PDCAAS) compared to soy protein (Cao X. et al., 2025). YP is also free from lactose found in whey protein or allergens found in soy, and thus better tolerated by individuals with lactose intolerance or soy allergy. Unlike whey protein, YP is derived from microorganisms, thus also meeting the vegan dietary requirements. Moreover, its production process is highly efficient in energy and resource use compared to that of animal- and plant-based proteins, making it a more sustainable protein-rich alternative to whey and soy protein (Sá et al., 2020). As global demand for protein continues to rise, alternative sources like single-cell proteins from yeast have a significant potential for supporting the health of the aging population.

Although clinical trials are the gold standard for proving efficacy, they are poorly suited for assessing intervention effects on the gut microbiome. This is largely due to the substantial variation in microbiome composition both between and within individuals, which complicates the identification of treatment-induced changes (Lavelle et al., 2015). Additionally, a specific challenge with microbial metabolites is that they are often difficult to track due to their rapid absorption or utilization in the body (Ruppin et al., 1980; Delcour et al., 2016). These drawbacks can be overcome using the *ex vivo* SIFR^®^ technology (Systemic Intestinal Fermentation Research), which is a high-throughput, miniaturized, bioreactor-based model that accurately cultivates the gut microbiome of test subjects under controlled conditions, enabling mechanistic research to establish cause-and-effect relationships. Importantly, this technology has been validated to provide clinically predictive insights in both gut microbiome modulation (Van den Abbeele et al., 2023c) as well as subsequent effects on gut barrier integrity and immune function (Van den Abbeele et al., 2024). A key feature is its ability to simulate not only the colonic environment but also advanced upper gastrointestinal digestion and absorption processes (Deyaert et al., 2024), which is essential for studying complex foods or ingredients that are partially digestible (like proteins). While microbial responses are assessed within short time frames (24–48 h) in this *ex vivo* model, these effects have been shown to reflect outcomes observed in clinical trials following long-term exposure.

This study aimed to assess how YP influences composition of aging people’s microbiota and its metabolite production, as well as its subsequent effects on gut barrier integrity and immune function. We used *ex vivo* SIFR^®^ technology (simulating male adults aged 50–65 years) combined with a co-culture of epithelial and immune cells. To benchmark the effects of YP, we also tested a no-substrate control (NSC) and two other proteins: soybean protein isolate (SPI), and whey protein isolate (WPI). Our results underscore the potential of proteins to promote healthy aging by influencing the gut metabolome, reducing inflammation and promoting barrier integrity, with the specific protein source shaping the overall effect.

## 2 Materials and methods

### 2.1 Test compounds

Yeast protein was provided by Angel Yeast Co. Ltd. (Yichang, China), while SPI and WPI were purchase, respectively, from Shansong Biological Products Co. Ltd. (Linyi, China) and Fonterra Co-operative Group Limited (New Zealand). The amino acid composition of the three protein supplements has been recently published (Cao X. et al., 2025). According to this study, the true protein digestibility (TPD) of YP (84.87%) was lower than that of WPI (96.13%) and SPI (94.87%). The same publication also provides true ileal digestibility (TID) values for the individual amino acids in each protein.

### 2.2 Fecal donor selection criteria

Fresh fecal samples were collected according to a procedure approved by the Ethics Committee of the University Hospital Ghent (reference number BC-09977; approval date 13/4/2021). This involved participants signing an informed consent in which a fecal sample was donated for the study according to procedures that involve anonymization. Inclusion criteria were: 50–65 years of age, male, no antibiotic use in the past 3 months, no gastrointestinal disorders (cancer, ulcers, inflammatory bowel disease (IBD)), non-smoking, alcohol consumption < 3 units/d and 20 < BMI < 25. Six test subjects were enrolled with an average age of 61 (±4 years).

Freshly collected samples were converted to a fecal slurry using anaerobic phosphate-buffered saline (PBS). After homogenization, large particles were removed by gentle centrifugation (500 × *g*, 2 min) prior to being used in the bioreactor management device.

### 2.3 *Ex vivo* intestinal fermentation assay (SIFR^®^) design

**FIGURE 1:**
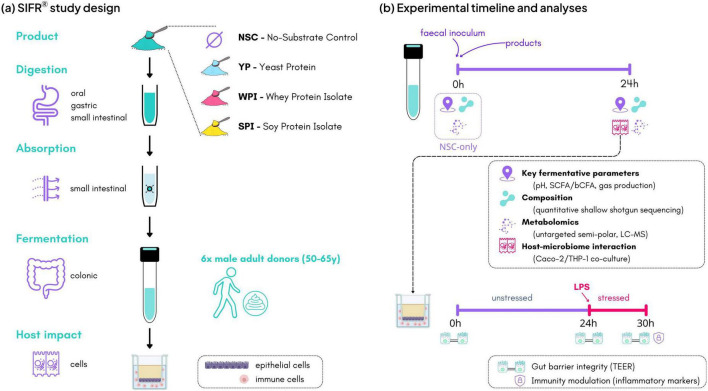
The impact of proteins of three different sources (YP, WPI, SPI; tested at 40 g/day) on the gut microbiome, gut barrier integrity and immune modulation was assessed using the *ex vivo* SIFR^®^ technology. **(a)** Study design of full gastrointestinal simulation and host-microbiome interaction assay along with **(b)** timeline and analysis. YP, yeast protein; WPI, whey protein isolate; SPI, soy protein isolate.

Upper gastrointestinal digestion and absorption and colonic fermentation were investigated using the SIFR^®^ technology as previously described (Van den Abbeele et al., 2023a,c). Four study arms were tested. Besides proteins of three different sources (YP, WPI, SPI), each tested at an equivalent of 40 g/day (resulting in a final colonic test concentration of 40 g/L, of which around 85% is digested along the upper GIT simulation as described in section “2.1 Test compounds”), an unsupplemented parallel control (NSC) was also included (Figure 1a). The test dose was chosen based on previously recommended daily intake of protein supplements in the elderly (Chapman et al., 2021). The NSC served as reference condition for evaluating treatment effects given that it is identical to protein treatments (same microbiome, same nutritional medium), except for the presence of proteins. NSC samples were run in technical triplicate for each test subject and monitored for key fermentative parameters at 24 h, with coefficients of variation below 3% confirming the high technical reproducibility of the SIFR^®^ technology (Van den Abbeele et al., 2023c). In total, each of the six fecal samples was tested with all three protein treatments and NSC (in triplicate), resulting in six tests per donor and a total of 36 individual bioreactors.

Previous studies showed that only around 10% of dietary protein reaches the colon (Bartlett and Kleiner, 2022). Digestibility of YP, WPI, and SPI has been previously evaluated using growing rats and INFOGEST model (Cao X. et al., 2025). Therefore, the test products or distilled H_2_O for NSC were subjected to oral, gastric and small intestinal digestion according to the INFOGEST 2.0 method (Brodkorb et al., 2019), performed as previously described by Van den Abbeele et al. (2023a). To ensure compatibility with colonic incubations, modifications were implemented such as simulation of small intestinal absorption, using a 3.5 kDa dialysis membrane to remove smaller peptides and amino acids (Van den Abbeele et al., 2023a). Further, at the start of the colonic incubations, individual fecal samples were processed in a bioreactor management device (Cryptobiotix, Ghent, Belgium) (Van den Abbeele et al., 2023c). Each bioreactor contained 5 mL of a blend of 2.0 mL small intestine-derived suspension, 2.5 mL nutritional medium (M0017, Cryptobiotix, Ghent, Belgium) and 0.5 mL of a fecal inoculum from a single donor (prepared as mentioned in section “2.2 Fecal donor selection criteria”). Bioreactors were sealed individually and rendered anaerobic. After preparation, bioreactors were incubated under continuous agitation (140 rpm) at 37 °C (MaxQ 6000, Thermo Scientific, Merelbeke, Belgium). Upon gas pressure measurement, samples were collected at 0 and 24 h for measurement of host-microbiome interactions, key fermentative parameters (pH, gas, SCFA and bCFA production), microbial composition and in-depth metabolite analysis (untargeted metabolite profiling) (Figure 1b).

### 2.4 Host-microbiome interaction assay

A coculture of Caco-2 (epithelial cells) and THP1-cells (immune cells) was used to assess the impact of fermented proteins on host health, according to a previously published protocol (Satsu et al., 2006; Van den Abbeele et al., 2024). Briefly, Caco-2 and THP1-cells were differentiated over 14 and 2 days, respectively, after which immune cells were covered with an epithelial layer in a permeable well insert (Figure 1a). Centrifuged colonic samples (5’ at 9,000 × *g*) were filtered (0.22 μm) prior to use in the host-microbiome interaction assay, which consisted of two phases (Figure 1b): (i) 24 h treatment to evaluate the impact of colonic samples (applied on the apical side of epithelial cells) on barrier integrity under unstressed conditions, and (ii) an additional 6 h incubation in presence of lipopolysaccharide (LPS) to evaluate effects on barrier integrity under stressed conditions, along with immunomodulatory effects (samples collected at 30 h). As LPS triggers a rapid and strong inflammatory response, a 6 h incubation was sufficient to elicit measurable stress-induced effects. Transepithelial electrical resistance (TEER) informed on gut barrier integrity and was measured at 0, 24 (before LPS addition), and 30 h (6 h after LPS addition). Immune effects were studied via cytokine and chemokine production using a Multiplex Luminex^®^ Assay kit on the MAGPix^®^ analyzer (CXCL10, IL-10, IL-1β, TNF-α). In addition to the colonic samples, the assay also contained four reference samples, i.e., (i) a blank that was not treated with any compounds, including LPS, throughout the experiment; (ii) a reference that after 24 h, was treated with LPS for additional 6 h; (iii), (iv) two additional references samples similar to (ii) that were, respectively also incubated with the anti-inflammatory compounds dexamethasone (D) and hydrocortisone (HC) throughout the experiment.

### 2.5 Key fermentative parameters

Short-chain fatty acid (acetate, propionate, butyrate and valerate) and branched-chain fatty acids (bCFA; sum of isobutyrate and isovalerate) were determined via gas chromatography with flame ionization detection (GC-FID), after extraction in diethyl ether, as previously described (Van den Abbeele et al., 2023b). Briefly, samples were acidified with sulfuric acid, after which an excess of sodium chloride was added along with internal standard (2-methylhexanoic acid) and diethyl ether. Upon homogenization and separation of the diethyl ether layer, extracts were analyzed using a Trace 1300 chromatograph (Thermo Fisher Scientific, Merelbeke, Belgium) equipped with a Stabilwax-DA capillary GC column, a flame ionization detector, and a split injector using nitrogen gas as the carrier and makeup gas. Finally, pH was measured using an electrode (Hannah Instruments Edge HI2002, Temse, Belgium).

### 2.6 Taxonomic microbiota analysis by quantitative shotgun sequencing

Quantitative insights were obtained by correcting proportions (%; shotgun sequencing) with total counts (cells/mL; flow cytometry), resulting in estimated cells/mL of different phyla, families and species.

First, DNA was extracted from a bacterial cell pellet (centrifugation of 1 mL sample for 5’ at 9,000 × *g*) via the SPINeasy DNA Kit for Soil (MP Biomedicals, Eschwege, Germany), according to manufacturer’s instructions. Subsequently, a library was prepared using the Nextera XT DNA Library Preparation Kit (Illumina, San Diego, CA, United States) and IDT Unique Dual Indexes (total DNA input, 1 ng). A proportional amount of Illumina Nextera XT fragmentation enzyme was added to fragment genomic DNA. Libraries were constructed, purified, and quantified as previously described (Van den Abbeele et al., 2023c), then sequenced on an Illumina Nextseq 2000 platform 2x150 base pairs. The CosmosID-HUB Microbiome Platform (CosmosID Inc., Germantown, MD, United States; accessed on 6 June 2024) was used to convert unassembled sequencing reads to relative abundances (%) (Hasan et al., 2014; Agarwal et al., 2022). To determine total counts, samples were diluted in PBS, followed by cell staining with SYTO 16 at a concentration of 1 μM, and counted via a BD FACS Verse flow cytometer (BD, Erembodegem, Belgium).

### 2.7 Untargeted metabolite profiling

Liquid chromatography-mass spectrometry (LC-MS) analysis was carried out on a Thermo Scientific Vanquish LC coupled to Thermo Q Exactive HF MS (Thermo Scientific), using an electrospray ionization source, both in negative and positive ionization mode. Ultra Performance Liquid Chromatography (UPLC) was performed applying a slightly modified version of the protocol described by Doneanu et al. (2011). Peak areas were extracted using Compound Discoverer 3.1 (Thermo Scientific), along with a manual extraction based on an in-house library using Skyline 21.1 (MacCoss Lab Software, University of Washington, Seattle, WA, United States) (Adams et al., 2020). Identification of compounds was performed at different levels. The analysis focused on level 1 and 2a metabolites, ensuring correct annotation, i.e., level 1 (retention times (compared against in-house authentic standards), accurate mass (with an accepted deviation of 3ppm), and MS/MS spectra), level 2a (retention times and accurate mass).

### 2.8 Data analysis

All analyses were performed using R (version 4.2.2 www.r-project.org; accessed on 18 November 2024). This software was used to make principal component analysis (PCA), bar charts, violin plots and heat maps. For the PCA, the FactoMineR package was used (Husson et al., 2022). Significance of treatment effects were assessed via repeated measures ANOVA analyses (based on paired testing among the six test subjects), with *p*-value correction according to Benjamini and Hochberg (1995). “*” indicates the significance of a potential treatment effect versus the NSC. “$” indicates a significant difference between WPI, SPI and YP, while “&” highlights a significant difference between SPI and WPI.

Heat maps present log_2_-transformed fold changes for the different treatments compared to the parallel control arm (NSC). This way, when a microbial group or metabolite is increased by a given treatment, a positive value is displayed, while negative values reveal a decrease. Significant differences compared to NSC are highlighted in bold.

For the statistical analysis of microbial composition, three measures were taken. First, the analysis was performed on log_2_-transformed values. Second, a value of a given taxonomic group below the limit of detection (LOD) was considered equal to the LOD, as described before (Van den Abbeele et al., 2023c). Finally, a threshold was set to retain the 150 most abundant species, to avoid major *p*-value corrections. These species provided comprehensive insights as they covered on average 89.7% of the total abundances of a given sample.

## 3 Results

### 3.1 The study cohort covered enterotypic differences described for human adults

The current study assessed the impact of protein supplements derived from three different sources on the gut microbiota of male adults aged 50–65 years. Individuals aged 50–65 years show less pronounced age-related decline in physiological functions, immune systems, and gut functions compared to older individuals. Their functions may be weakening but still have modifiable and restorable potential, making protein interventions more meaningful (Aronson, 2020). However, this age range partially coincide with the menopausal transition in females which can significantly impact the gut microbiome (Liaquat et al., 2025). Given the size of the cohort, a single sex was selected for the study to minimize complexity from sex-related variations in the cohort.

Human fecal microbiota composition markedly differed between the six male adult test subjects aged 50–65 years at baseline (Supplementary Figure 1). PCA revealed that most interpersonal variation was driven by elevated levels of *Prevotella* (subjects 1, 4, and 5), *Bacteroides*, *Phocaeicola* (subject 6) and *Faecalibacterium*, *Ruminococcus*_E, *Blautia*_A (subjects 2 and 3). These differences align with the stratification of human gut microbiota according to the concept of enterotypes (Costea et al., 2018), which distinguishes three types (*Prevotella, Bacteroides*, and Firmicutes) based on the abundance of the respective taxa. Since our cohort included representatives of each enterotype, the observed interpersonal variation supported the relevance and representativeness of the findings for the broader population.

### 3.2 The proteins stimulated SCFA and bCFA, promoted gut barrier integrity and exerted anti-inflammatory effects in a protein-specific manner

**FIGURE 2:**
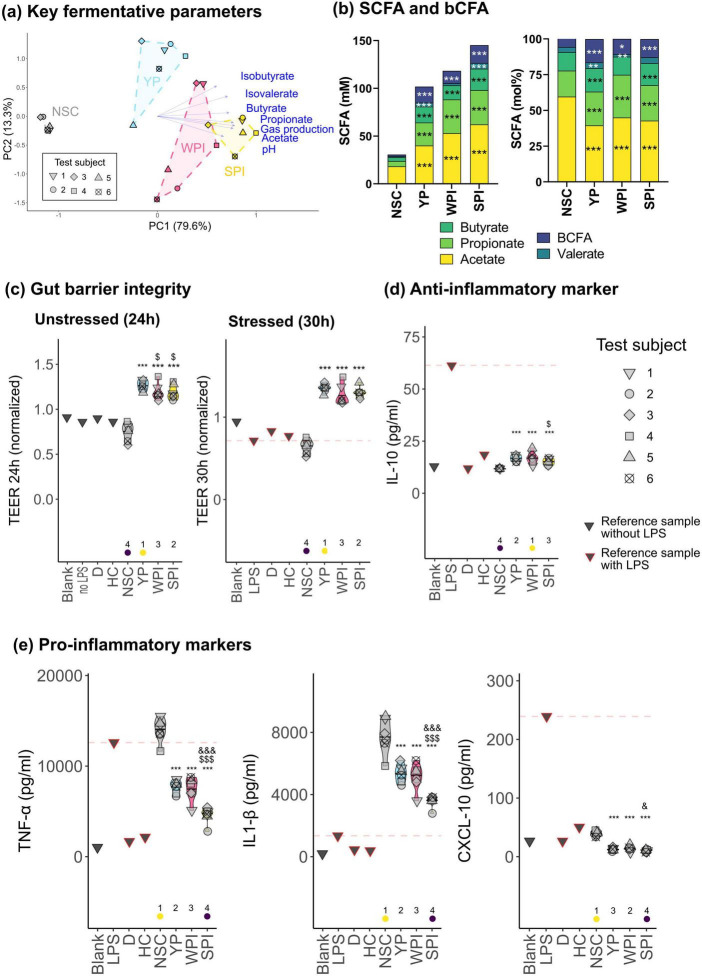
The proteins (YP, WPI, SPI) stimulated short-chain fatty acid (SCFA) and branched-chain fatty acids (bCFA), promoted barrier integrity and exerted anti-inflammatory effects in a protein-specific manner. **(a)** Principal component analysis (PCA) summarizing the impact on key fermentative parameters (pH, gas, SCFA, bCFA). A detailed representation of each parameter is provided in Supplementary Figure 2. **(b)** SCFA and bCFA, averaged across all test subjects, expressed as absolute (mM) or proportional levels (mol%). **(c)** Gut barrier integrity, both under unstressed (24 h) and lipopolysaccharide (LPS)-stressed conditions (30 h), measured as transepithelial electrical resistance (TEER) of the Caco-2 layer, normalized to the TEER value at 0 h. **(d)** Anti-inflammatory (IL-10) and **(e)** pro-inflammatory markers (TNF-α, IL1-β, CXCL-10). Statistical differences with no-substrate control (NSC) are indicated with * (0.10 < *p*_adjusted_ < 0.20), ** (0.05 < *p*_adjusted_ < 0.10) or *** (*p*_adjusted_ < 0.05). “$/$$/$$$” indicates differences between YP and WPI or SPI, while “$/$$/$$$” indicates difference between WPI and SPI. Ranks of averages per treatment are indicated, with the lowest value indicated in purple, and the highest in yellow. Four reference samples were also included in the visualization of **(c–e)**. The horizontal dash line indicates the value of the reference sample when treated with lipopolysaccharide (LPS).

Upon oral, gastric, small intestinal and colonic incubation, a first exploratory analysis of key fermentative parameters revealed that for all six different test subjects, treatments with YP, WPI, and SPI are positioned to the right side of the PCA associated with higher production of gases, SCFA and bCFA (Figure 2a). All proteins indeed significantly increased acetate, propionate, butyrate, isobutyrate, isovalerate and gas production (Supplementary Figure 2).

While the PCA highlighted some interindividual differences, as evidenced by the spread of samples within treatment group, it also highlighted protein-specific effects that were generally consistent across test subjects (samples of different treatments did not overlap) (Figure 2a). Protein-specific effects were that (i) SPI exerted the strongest stimulation of total SCFA and bCFA production, and (ii) WPI and YP displayed a high proportion (mol %) of acetate/propionate and butyrate/bCFA, respectively, and thus higher specificity toward stimulating these respective metabolites (Figure 2b). In addition, YP resulted in remarkably low gas production (Supplementary Figure 2B). Indeed, when gas production was normalized against total SCFA levels, YP produced 27% and 19% less gases compared to WPI and SPI, respectively (Supplementary Figure 2K).

A host-microbiome interaction assay was performed to assess the impact of protein fermentation on gut barrier integrity and immune response. Barrier function was assessed via TEER, a widely accepted indicator of epithelial integrity (Srinivasan et al., 2015), while immune modulation was evaluated by measuring an anti-inflammatory (IL-10) and three pro-inflammatory markers (TNF-α, IL1-β and CXCL-10). First, the LPS reference sample revealed that the 6 h treatment with LPS markedly decreased TEER (Figure 2c) and strongly induced the release of all four immune markers measured in the experiment (IL-10, TNF-α, IL1-β, and CXCL-10) (Figures 2d, e). As anticipated, treatment with the anti-inflammatory compounds D and HC partially reversed the impact of LPS on TEER and reduced the levels of the immune markers to the similar levels of the blank sample. Further, the treatment of the cells with the colonic samples revealed that all proteins, especially YP, significantly increased TEER, thus lowering gut permeability and enhancing barrier integrity compared to NSC, both under unstressed (24 h) and stressed conditions (30 h, including 6 h LPS treatment) (Figure 2c). Especially, under the stressed condition, the effect of the proteins exceeded the control samples D and HC. Importantly, there was a strong positive correlation between elevated SCFA levels and promotion of gut barrier integrity for the colonic samples (Supplementary Figure 3). All proteins also significantly stimulated the anti-inflammatory cytokine IL-10, while the levels of pro-inflammatory markers TNF-α, IL1-β, and CXCL-10 were lowered, especially with SPI (Figures 2d, e). Again, SCFA levels strongly correlated with changes in these markers (positively with IL-10 and negatively with TNF-α, IL1-β, and CXCL-10) (Supplementary Figure 4).

### 3.3 Proteins stimulated microbial diversity and modulated microbiota composition in a protein-specific manner

**FIGURE 3:**
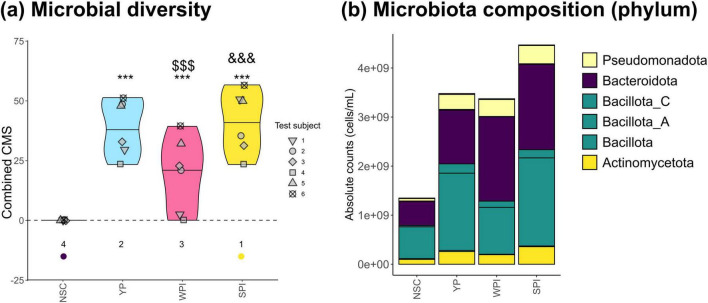
The proteins (YP, WPI, SPI) stimulated microbial diversity and modulated microbiota composition in a protein-specific manner. **(a)** The impact on microbial diversity (combined CMS) and **(b)** microbial composition at phylum level (cells/mL). Statistical differences with no-substrate control (NSC) are indicated with * (0.10 < *p*_adjusted_ < 0.20), ** (0.05 < *p*_adjusted_ < 0.10) or *** (*p*_adjusted_ < 0.05). “$/$$/$$$” indicates differences between YP and WPI or SPI, while “$/$$/$$$” indicates difference between WPI and SPI. Ranks of the average values per treatment are indicated at the bottom, with the lowest average being indicated in purple, and the highest in yellow. CMS, community modulation score.

To understand which gut microbes were responsible for the changes in key fermentative parameters, microbiota composition was analyzed via quantitative shallow shotgun sequencing. First, the combined community modulation score (CMS) was calculated. This index was recently developed to overcome biases inherent in traditional diversity metrics, which can underestimate diversity due to changing detection limits when microbial load increases (Tintoré et al., 2024). All proteins, especially YP and SPI, increased the combined CMS, thus displaying a positive impact on microbial diversity, suggesting the stimulation of a wide range of gut microbes (Figure 3a). In contrast, traditional diversity indices failed to capture these effects, likely due to their sensitivity to shifts in total cell counts (Supplementary Figure 5).

A first visualization of microbial composition at phylum level revealed that all proteins promoted Actinomycetota, Pseudomonadota and particularly Bacillota and Bacteroidota (formerly Actinobacteria, Proteobacteria, Firmicutes and Bacteroidetes, respectively) (Figure 3b). Again, protein-specific effects were noted: SPI exerted strongest absolute stimulation of the various phyla, while WPI and YP had a high specificity for Bacteroidota and Bacillota, respectively.

**FIGURE 4:**
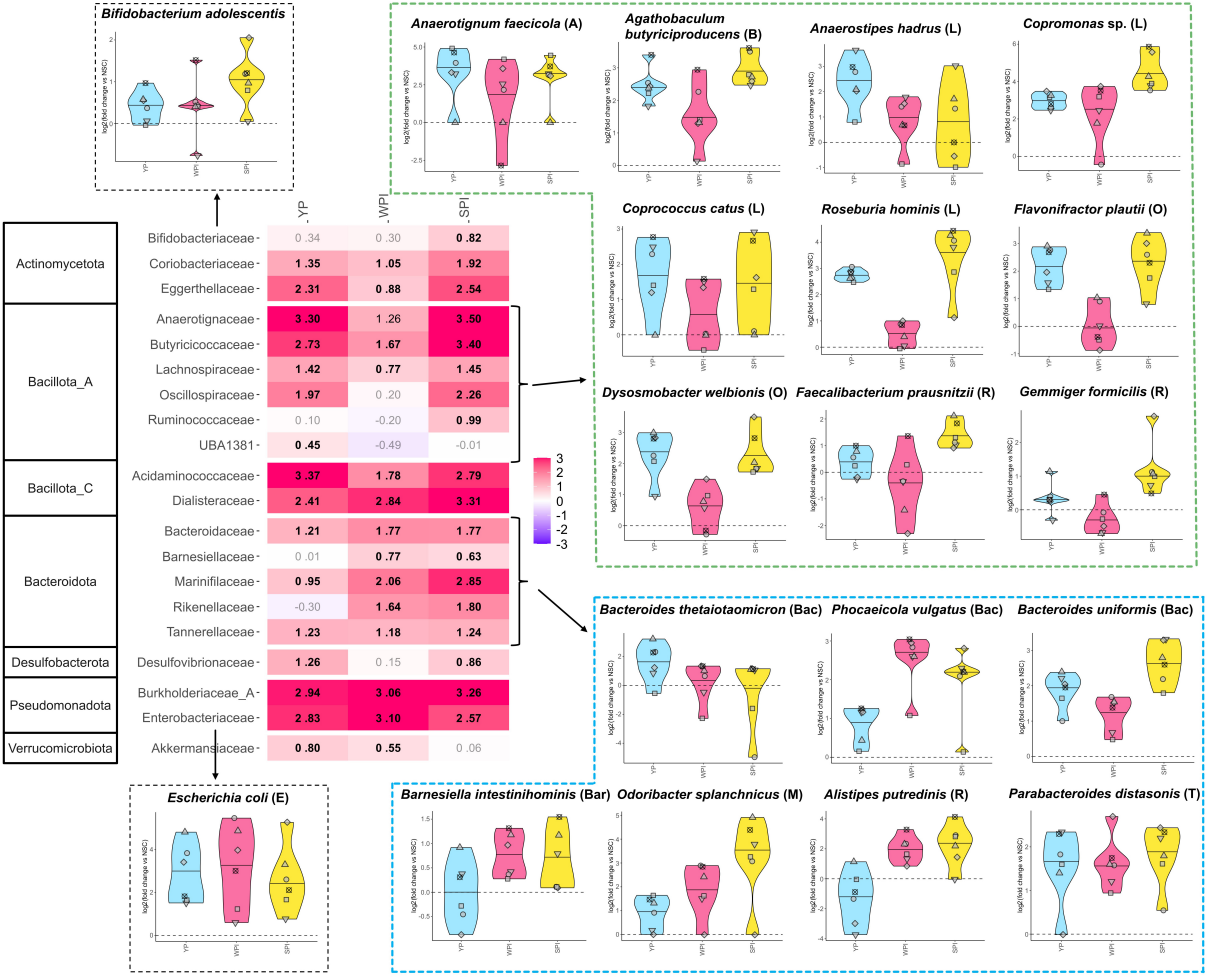
The proteins (YP, WPI, SPI) stimulated specific microbial families and species in a protein-specific manner. Heatmap based on families that were significantly [false discovery rate (FDR) = 0.20] affected by any of the treatments, expressed as log_2_ (treatment/NSC), averaged over all test subjects. Values indicated in bold show significant increases (>0) or decreases (<0). Corresponding phyla are indicated on the left for each family. A selection of key species belonging to the affected families, again expressed as log_2_ (treatment/NSC) is presented in dedicated violin plots. Thus, the value of the NSC is set at 0 and marked by a horizontal dashed line. A detailed representation of all significantly affected species is provided in Supplementary Figure 6.

The strong stimulation of Bacillota by SPI and YP were generally quite similar at higher phylogenetic resolution (family and species level; Figure 4), followed from potent increases of butyrate-producing communities belonging to Anaerotignaceae (*Anaerotignum faecicola*), Butyricicoccaceae (*Agathobaculum butyriciproducens*), Lachnospiraceae (*Anaerostipes hadrus* (higher increase for YP), *Copromonas* sp. (higher increase for SPI), *Coprococcus catus* and *Roseburia hominis*), Oscillospiraceae (*Dysosmobacter welbionis* and *Flavonifractor plautii*) and Ruminococcaceae [*Faecalibacterium prausnitzii* and *Gemmiger formicilis* (higher increase for SPI)]. In line with their metabolic capacity, positive correlations were established between these families and butyrate levels (Supplementary Figure 7; Louis and Flint, 2017).

Further, within the Bacteroidota, all proteins similarly increased Tannerellaceae (*Parabacteroides distasonis*) and Bacteroidaceae, which was due to protein-specific effects on different species, i.e. *Bacteroides thetaiotaomicron* (YP), *Phocaeicola vulgatus* (WPI) or *Bacteroides uniformis* (SPI). The stronger stimulation of Bacteroidota by SPI and WPI followed from stronger increases of Barnesiellaceae [*Barnesiella intestinihominis* (WPI, SPI)], Marinifilaceae [*Odoribacter splanchnicus* (SPI)] and Rikenellaceae [*Alistipes putredinis* (WPI, SPI)]. Importantly, Bacteroidaceae, Tannerellaceae and Rikenellaceae displayed positive correlations with propionate levels (Supplementary Figure 7), in line with their role as prominent propionate producers of the gut microbiota (Louis and Flint, 2017). Finaly, the mild stimulation of Actinomycetota and Pseudomonadota followed from increases in Bifidobacteriaceae (SPI) and Enterobacteriaceae (all proteins), respectively.

### 3.4 Proteins modulated the gut metabolome in a protein-specific manner, including health-related amino acid derivatives

To evaluate the production of protein-derived metabolites beyond traditionally studied key fermentative parameters, untargeted metabolite profiling was performed (LC-MS). First, all detected amino acids were efficiently consumed in the no-substrate control (NSC) between 0 and 24 h, confirming the proteolytic activity of the microbiota of each test subject (Supplementary Figure 8). Further, despite the digestion/absorption along the upper GIT and fermentation by bacteria (processes that lower amino acid levels), proteins still elevated the levels of all essential amino acids upon 24 h of fermentation (isoleucine, leucine, lysine, methionine, phenylalanine, tryptophan and valine), compared to NSC.

In-depth analysis revealed that 91 metabolites increased for at least four of the six test subjects upon 24 h of colonic incubation in any of the study arms (compared to NSC at 0 h), suggesting their production by gut microbes or presence in one of the proteins. A total of 78 of these metabolites were significantly affected (false discovery rate = 0.20) (Supplementary Figure 9).

**FIGURE 5:**
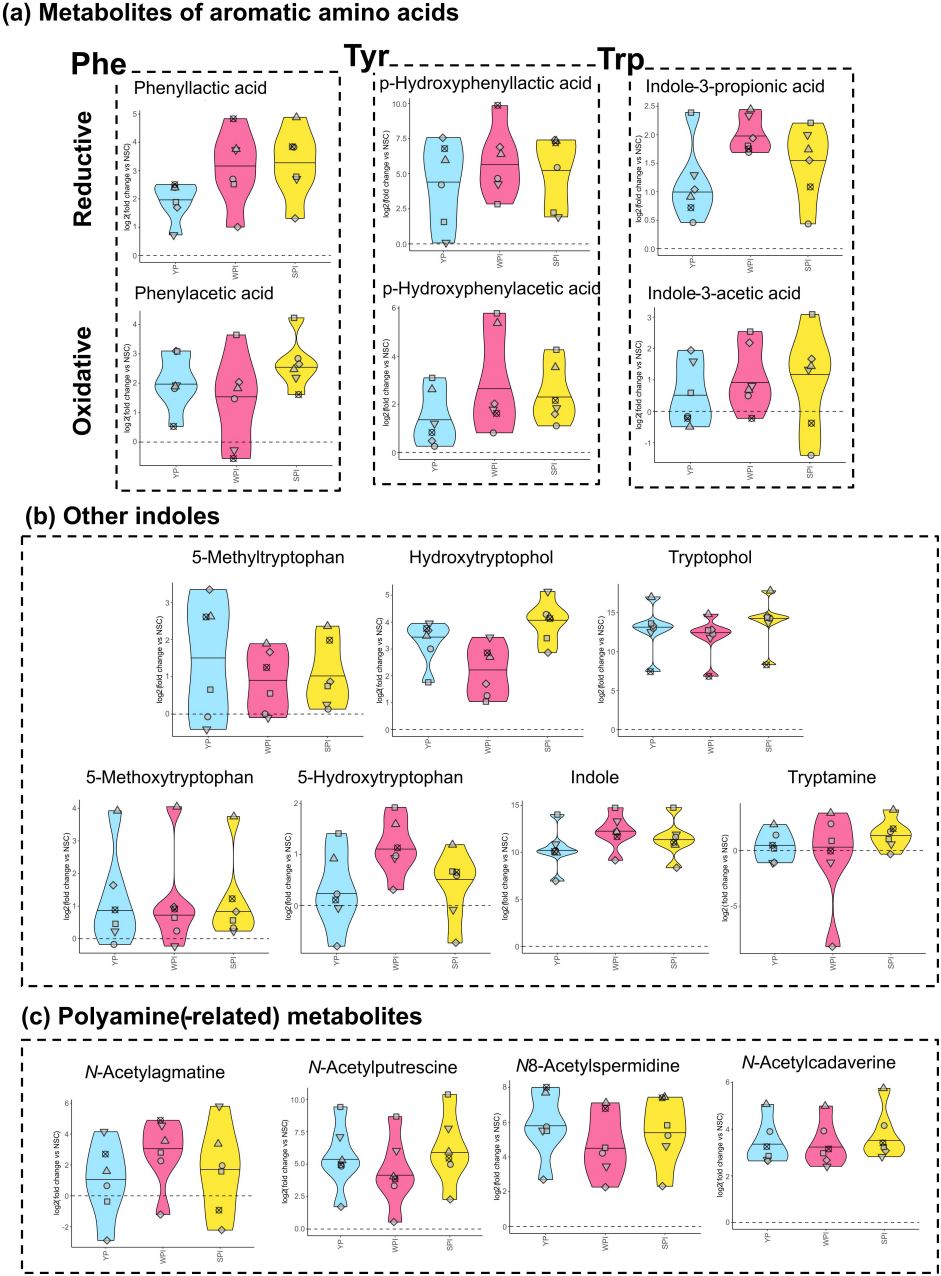
The proteins (YP, WPI, SPI) significantly stimulated a spectrum of amino acid-related metabolites in a protein-specific manner. The metabolites are presented in dedicated violin plots, expressed as log_2_ (treatment/NSC), for each of the six 50–65 years male adults. Thus, the value of the NSC is set at 0 and marked by a horizontal dashed line. **(a)** Metabolites involved in the reductive and oxidative pathways of aromatic amino acid fermentation. **(b)** Other tryptophan-derived indoles and **(c)** polyamine-related metabolites. A detailed representation of all impacted metabolites is provided in Supplementary Figure 8.

The test products had a strong and protein-specific impact on the gut metabolome. A first class of amino acid-derived metabolites involved metabolites of the reductive and oxidative pathways of the aromatic amino acids phenylalanine (Phe), tyrosine (Tyr) and tryptophan (Trp) (Figure 5a). In the reductive pathway, two aromatic lactic acids (3-phenyllactic acid, *p*-hydroxyphenyllactic acid) were boosted by all proteins, with the third aromatic lactic acid (indole-3-lactic acid) not being detected. Instead, its downstream metabolite, indole-3-propionic acid (IPA), showed a substantial increase across all three test products, with the most pronounced effect observed in response to WPI. In addition to the reductive pathway, proteins also enhanced the production of three metabolites from the oxidative pathway: phenylacetic acid, *p*-hydroxyphenylacetic acid, and indole-3-acetic acid (IAA). Furthermore, proteins stimulated the production of several other indole-related metabolites (Figure 5b). Notably, WPI led to higher levels of IPA, indole, and 5-hydroxytryptophan, while SPI more strongly promoted tryptophol [or 3-(2-hydroxyethyl)indole] and hydroxytryptophol.

Another distinct class of metabolites promoted by the proteins were acetylated polyamines. These included *N*-acetylputrescine and its derivative N8-acetylspermidine, both derived from arginine, as well as the lysine-derived *N*-acetylcadaverine [or *N*-(5-aminopentyl)acetamide] (Figure 5c). Additionally, acetylated agmatine, a precursor of putrescine and spermidine, was notably increased for several donors by YP, SPI, and especially by WPI. Additional metabolites were most strongly promoted by YP [cephalotaxine, pipecolinic acid (PIPA) and kynurenine (KYN)], SPI (lathyrol and 12,13-DiHOME) or WPI [hydroxyisocaproic acid (HICA) and trimethylamine *N*-oxide (TMAO)] (Supplementary Figure 10). Further, a large number of *N*-acetylated amino acids were promoted by all three protein sources, with the strength of the effects also often depending on the protein sources (Supplementary Figure 11).

Finally, while not discussed in the scope of this study, a spectrum of other amino acid-related metabolites was impacted by the proteins including, amongst many others, 3-aminoisobutanoic acid (BAIBA), γ-aminobutyric acid (GABA) as well as several oligopeptides (Supplementary Figure 9).

## 4 Discussion

Despite the consistency in high-level outcomes, distinct protein-specific effects were uncovered. First, all three protein sources had a positive impact on microbial diversity (based on the CMS) which usually declines with age, primarily due to reductions in beneficial species such as *Bifidobacterium* and certain members of Firmicutes (Bosco and Noti, 2021). This effect of the proteins is also consistent with a previous *in vivo* study showing correlations between high protein intake and increased intestinal diversity in elderly men (Farsijani et al., 2022).

All three proteins boosted Bacteroidota with the strongest stimulation observed with WPI, likely explaining the highly specific increase in propionate production (Louis and Flint, 2017). On the other hand, YP and SPI most strongly boosted the Bacillota phylum due to potent increases of a broad range of butyrogenic species including *Anaerotignum faecicola* [related to *A. aminivorans* which is capable of producing butyrate from L-threonine (Ueki et al., 2017)]*, Agathobaculum butyriciproducens* (Ahn et al., 2016), *Dysosmobacter welbionis* (Le Roy et al., 2022)*, Flavonifractor plautii* (Rodriguez-Castaño et al., 2020), *Anaerostipes hadrus* (Louis and Flint, 2017), *Faecalibacterium prausnitzii* (Allen-Vercoe et al., 2012) and *Roseburia hominis* (Patterson et al., 2017) abundances. This effect is especially of interest as several of the aforementioned taxa, including *Faecalibacterium*, *Roseburia*, *Coprococcus and Gemmiger*, have been previously proposed as anti-aging microbial markers in both humans and nonhuman primates (Sang et al., 2022). The anti-aging effects of these taxa are, at least in part, attributed to their ability to produce butyrate. Butyrate, a short-chain fatty acid and the preferred energy source for colonocytes, plays a critical role in maintaining colon health (Morrison and Preston, 2016). This mechanism contributes to the positive impact of all protein sources, particularly YP, on epithelial integrity. Thus, YP may be particularly effective in supporting gut barrier integrity, a function that typically tends to decline with age (Branca et al., 2019). In addition, YP supplementation may also be relevant in individuals with diseases related to gut barrier dysfunction such as IBD. Additionally, the increased SCFA production with the proteins correlated with increased release of anti-inflammatory IL-10 and decreased secretion of pro-inflammatory markers. These effects are especially relevant for the elderly population, as aging is commonly linked to chronic inflammation (Li et al., 2023) and a decline in intestinal epithelial integrity (Conway et al., 2025). Furthermore, SCFAs also alleviate systemic inflammaging through gut-organ axes, providing benefits that extend beyond the gut. For example, they have been shown to improve acute lung injury (Hildebrand et al., 2023) and neuroinflammation associated with neurodegenerative diseases (Kalyanaraman et al., 2024). SCFAs also contribute to improved metabolic health and reduced obesity risk by enhancing insulin sensitivity (Pham et al., 2024) and promoting the secretion of satiety hormones (Chambers et al., 2018). Additionally, butyrate exhibits therapeutic potential in oncology, partly due to its ability to inhibit histone deacetylases, thereby suppressing tumor cell proliferation (Sun et al., 2024; Nshanian et al., 2025). Recently, butyrate has also been associated with improved cognitive function in older adults (Tu et al., 2025). Therefore, stimulating SCFA production, particularly by substrates like YP that strongly enhance butyrate, may offer significant health benefits for the aging population.

Beyond the benefits of SCFAs, *Faecalibacterium prausnitzii* is known to exert potent anti-inflammatory effects through the production of a specific protein called Microbial Anti-inflammatory Molecule (MAM) (Breyner et al., 2017). The strongest stimulation of *F. prausnitzii* observed with SPI may likely contribute to the most pronounced reduction in pro-inflammatory cytokines observed for this protein source. This was further supported by significant reverse correlations between *F. prausnitzii* and pro-inflammatory markers IL-1β and TNF-α (Supplementary Figure 12). Therefore, although SPI induced a smaller increase in gut barrier integrity compared to YP, it may be particularly beneficial in conditions with active intestinal inflammation that requires chronic suppression such as autoimmune diseases.

Moreover, untargeted metabolite profiling provided great insights into how proteins impact the gut metabolome, well beyond short-chain fatty acids. This was enabled by the use of the *ex vivo* SIFR^®^ technology that allowed for quantification of the complete gut microbial metabolome. In contrast, *in vivo* studies (such as the recent mice study with the same proteins (Zhou et al., 2024)) typically rely on fecal samples, i.e., excretion products of the body from which many microbial metabolites have already been removed given their rapid absorption or utilization *in situ* (Ruppin et al., 1980; Delcour et al., 2016).

Various indoles derived from tryptophan catabolism were elevated by all three protein sources. Indoles have been associated with extending healthspan and lifespan (Sonowal et al., 2017; Cao Z. et al., 2025), primarily via activation of the aryl hydrocarbon receptor (AhR), a conserved receptor crucial for regulating immune responses. A notable example is indole-3-propionic acid (IPA), a gut microbiota-derived compound that exhibits potent antioxidative and anti-inflammatory properties. Similar to SCFAs, IPA can enhance gut barrier integrity by increasing the expression of tight junction proteins and mucins, inhibits the release of pro-inflammatory cytokines and suppresses NF-κB signaling to reduce intestinal inflammation (Zhao et al., 2019; Li et al., 2021). IPA readily crosses the intestinal epithelial barrier and enters systemic circulation, allowing it to exert antioxidative and anti-inflammatory effects in other organs. For example, IPA improves cardiovascular and metabolic health, promotes muscle growth, and prevents lung infections and kidney injury (Jiang et al., 2022). Importantly, probiotic-induced IPA production has been shown to exert neuroprotective effects in elderly adults, thus supporting neural and cognitive function (Kim et al., 2023). Other neuroactive indoles enhanced by the protein sources included 5-methoxytryptophan (5-MTP) as well as tryptamine, and tryptophol – both are functional analogues of the neurotransmitters melatonin and serotonin. Like IPA, 5-MTP has been extensively studied for its systemic health benefits, including its role in preventing cellular senescence (Wu et al., 2020; Wu, 2021). Further, all three proteins also promoted the production of indole itself which is synthesized exclusively through the action of the bacterial enzyme tryptophanase. It plays a critical role in inducing genes involved in intestinal epithelial barrier function and promoting the production of the anti-inflammatory cytokine IL-10 (Tennoune et al., 2022). Besides indoles, YP also strongly promoted kynurenine, a key tryptophan-derived metabolite in the kynurenine pathway. Activation of the kynurenine pathway has been shown to delay age-associated health decline in model organisms (Sutphin and Espejo, 2023). Altogether, these findings underscore the potential of microbial tryptophan catabolites in supporting systemic health and healthy aging.

Polyamines are another class of metabolites that help maintain the epithelial barrier function by regulating epithelial proliferation, enhancing autophagy which facilitates the removal of invading pathogens (Madeo et al., 2018; Nakamura et al., 2021). Besides, polyamines also exhibits anti-inflammatory and anti oxidative properties which have been associated with longevity (Soda, 2022). In particular, spermidine has been shown to extend lifespan in model organisms and improve healthspan by mitigating aging-associated pathologies, supporting cardiovascular health, and reducing the risk of neurodegenerative diseases (Eisenberg et al., 2016; Madeo et al., 2018). While polyamines can be directly obtained from dietary sources, gut microbes are capable of synthesizing polyamines from basic amino acids such as arginine and lysine (Xi et al., 2023). The elevated levels of acetylated forms of putrescine, spermidine, and cadaverine observed upon fermentation of all three proteins suggest the activation of polyamine production. Furthermore, the protein supplements increased agmatine, the precursor of putrescine and spermidine, which can interact with various receptors in the central nervous system and display neuroprotective effects (Xu et al., 2018).

Other notable metabolites promoted by the proteins potentially have health benefits that are relevant for the aging population, such as preventing muscle loss [α-hydroxyisocaproic acid (Lang et al., 2013)] and exerting neuroprotective effects [*N*-acetylleucine (Fields et al., 2023)]. In addition to stimulating the aforementioned beneficial metabolites, WPI strongly increased levels of TMAO, a compound associated with an elevated risk of cardiovascular diseases such as atherosclerosis, cardiac arrhythmia, hypertension as well as increased total mortality in elderly population (Lee et al., 2021; Fretts et al., 2022; Latif et al., 2025). This adverse effect is primarily attributed to pro-inflammatory properties of TMAO and its role in promoting cholesterol accumulation within arterial walls (Wang et al., 2011; Seldin et al., 2016). Therefore, the lower TMAO levels observed with SPI and YP indicate that these alternative protein sources may have fewer adverse side effects on cardiovascular health compared to the animal-derived WPI, which is typically richer in L-carnitine and choline, two precursors of TMAO.

Finally, YP resulted in a remarkably low increase in gas production compared to the other proteins. Excessive gas production can lead to bloating and abdominal discomfort (Kalantar-Zadeh et al., 2019). Thus, among the three proteins, YP may have the highest tolerability for human consumption.

While this study highlights the potential of specific protein sources to promote the proliferation of health-associated bacteria and the production of beneficial metabolites, it is important to recognize that protein-rich diets present a nuanced landscape with both potential health benefits and risk. Excessive protein intake, especially in Western-style diets, can lead to increased proteolytic fermentation, resulting in metabolites such as ammonia, sulfides and phenols, which have been associated with gastrointestinal disturbances and colonic inflammation (Yao et al., 2016; Diether and Willing, 2019; Wang et al., 2023). Elevated plasma levels of branched-chain amino acids (BCAAs) have been linked to metabolic syndrome and insulin resistance (Choi et al., 2024). However, circulating BCAA levels are more strongly influenced by overall dietary patterns and host metabolic status than by direct dietary intake (Merz et al., 2018; Solon-Biet et al., 2022). In our study, all protein sources elevated BCAA levels in the simulated colon, although the relevance of increased colonic BCAA to host health remains to be further explored. Indeed, proteins are rarely consumed in isolation, and co-ingested nutrients such as fibers may modulate fermentation dynamics and mitigate potential adverse effects. The source of protein also is an important influencing factor (Wang et al., 2023). Moreover, the current findings are based on *ex vivo* fermentation and coculture models, which – while validated to reflect clinical outcomes – cannot fully replicate *in vivo* physiology and host feedback mechanisms. Therefore, while the results presented in this study provide mechanistic insights, extrapolations to long-term health outcomes should be made with caution and ideally confirmed in human studies.

## 5 Conclusion

The results demonstrated that YP had comparable effects to WPI and SPI, two more commonly used protein sources, highlighting its strong potential as an alternative protein that supports healthy aging. YP contributed to the restoration of gut microbial diversity and stimulated the production of beneficial metabolites, including short-chain fatty acids (SCFAs), indoles, and polyamines. These benefits were further supported by its positive impact on intestinal epithelial integrity and its ability to reduce inflammation. Follow-up analyses assessing the expression of tight junction protein genes would offer additional mechanistic insights into how microbial fermentation of dietary proteins mediates improvements in gut barrier function. In addition, testing the individual effect of the different beneficial metabolites in the cell model could provide further insights into which metabolites mostly contribute to the improvements in barrier function. Further, it should also be noted that the predictive *ex vivo* SIFR^®^ colonic model has some limitations, especially the absence of host factors such as the lack of the host’s metabolic or immune feedback mechanisms. Therefore, further validation through human studies is necessary to prove the clinical efficacy of YP. Additionally, future research should include both male and female participants to evaluate the efficacy of the supplements in a broader population. Overall, protein supplements derived from sustainable sources should be considered promising dietary components in promoting healthy aging.

## Data Availability

The data presented in this study are publicly available. The data can be found here: https://www.ncbi.nlm.nih.gov, accession PRJNA1365657.
