# Supplementary material for: Yeast protein modulates metabolites derived from the human gut microbiota of older male adults ex vivo to strengthen gut barrier function and reduce inflammation
Source: Front Microbiol. 2026 Jan 6;16:1697734. doi: 10.3389/fmicb.2025.1697734 (PMC12816187; doi:10.3389/fmicb.2025.1697734)
Supplement: Supplementary file 1 [file Image_1.pdf]

Supplementary information

Yeast protein modulates metabolites derived from the human gut microbiota of older male adults *ex vivo* to strengthen gut barrier function and reduce inflammation

Pieter Van den Abbeele[1,*], Lam Dai Vu[1], Jonas Poppe[1], Ingmar A.J. van Hengel[1], Aurélien Baudot[1], Yan Zhang[2,3], Zhixian Chen[2,3] and Jun Yan[2,3]

[1]Cryptobiotix SA, Ghent, Belgium

[2] The Hubei Provincial Key Laboratory of Yeast Function, Angel Yeast Co. Ltd.,Yichang, China

[3] National Key Laboratory of Agricultural Microbiology, Angel Yeast Co. Ltd., Yichang, China

*** Correspondence:**
Corresponding Author
pieter.vandenabbeele@cryptobiotix.com

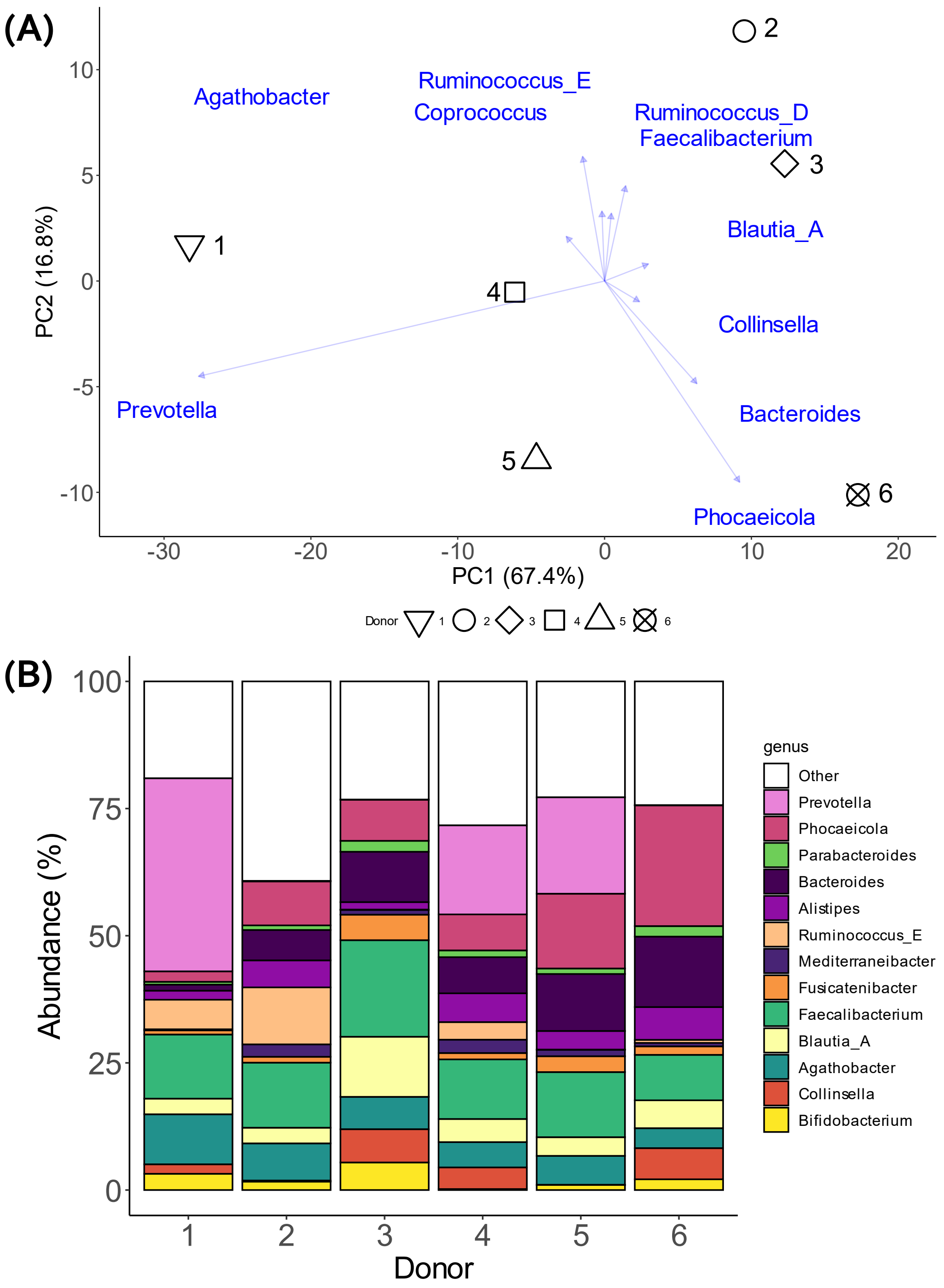

Figure S1. The fecal microbiota of 50-65y male adults (*n* = 6) covered clinically relevant interpersonal differences in line with the concept of enterotypes. (**A**) PCA based on centred abundances at genus level (%) demonstrating the variation across the faecal microbiota. (**B**) Abundances (%) of the key genera.

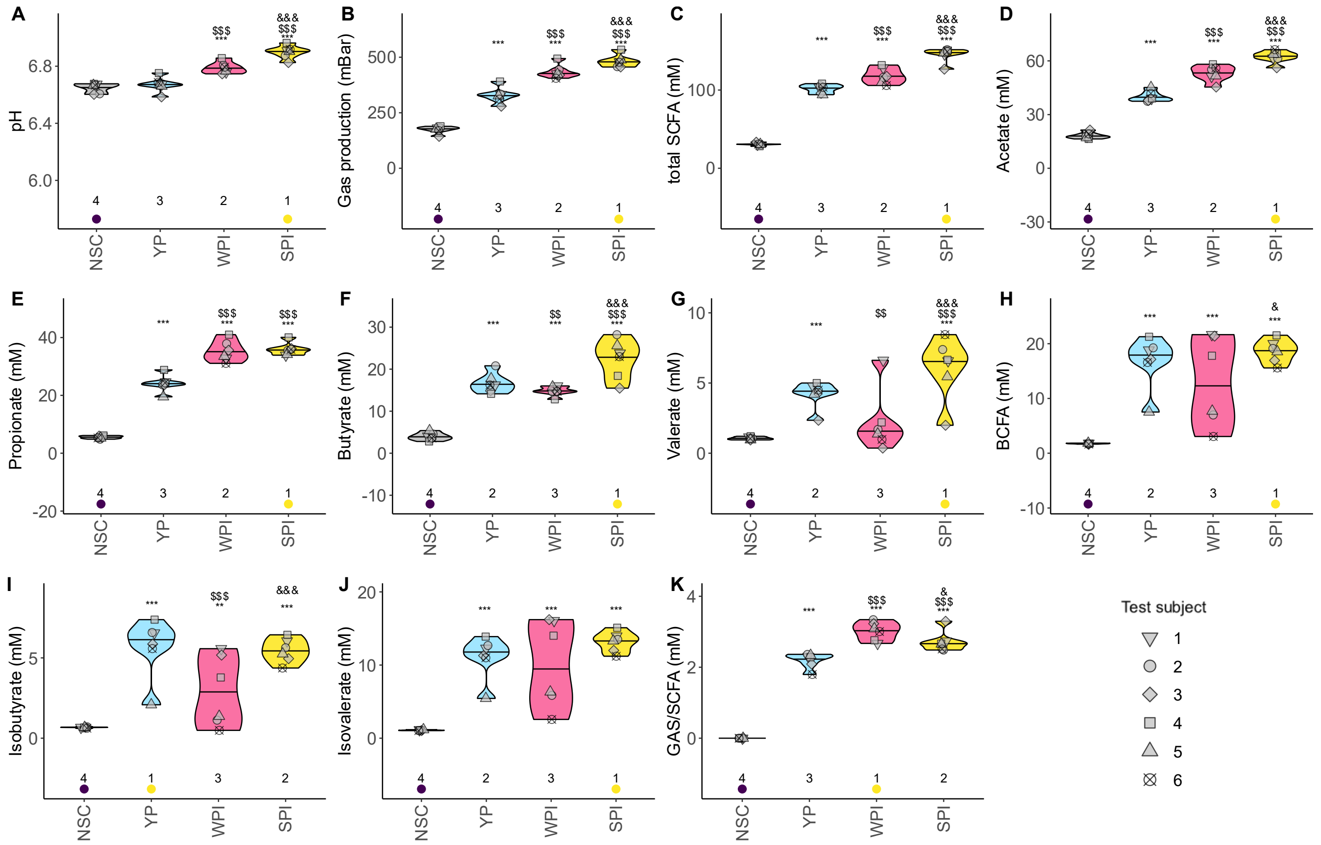

Figure S2. The proteins stimulated key fermentative parameters in a protein-specific manner. The impact on (**A**) pH, (**B**) gas production, (**C**) total SCFA, (**D**) acetate, (**E**) propionate, (**F**) butyrate, (**G**) valerate, (**H**) total bCFA, (**I**) isobutyrate, (**J**) isovalerate and (**K**) gas production, normalized against SCFA production compared to the NSC. Statistical differences with the NSC are indicated with * ($0.10 < p_{adjusted} < 0.20$), ** ($0.05 < p_{adjusted} < 0.10$) or *** ($p_{adjusted} < 0.05$). “$/$$/$$$” indicates differences between YP and WPI or SPI, while “$/$$/$$$” indicates differences between WPI and SPI. The ranks of the average values per treatment are indicated at the bottom of the figure, with the lowest average being indicated in purple, and the highest value in yellow. NSC: no-substrate control, YP: yeast protein, WPI: whey protein isolate, SPI: soy protein isolate, SCFA: short- chain fatty acids, bCFA: branched-chain fatty acids.

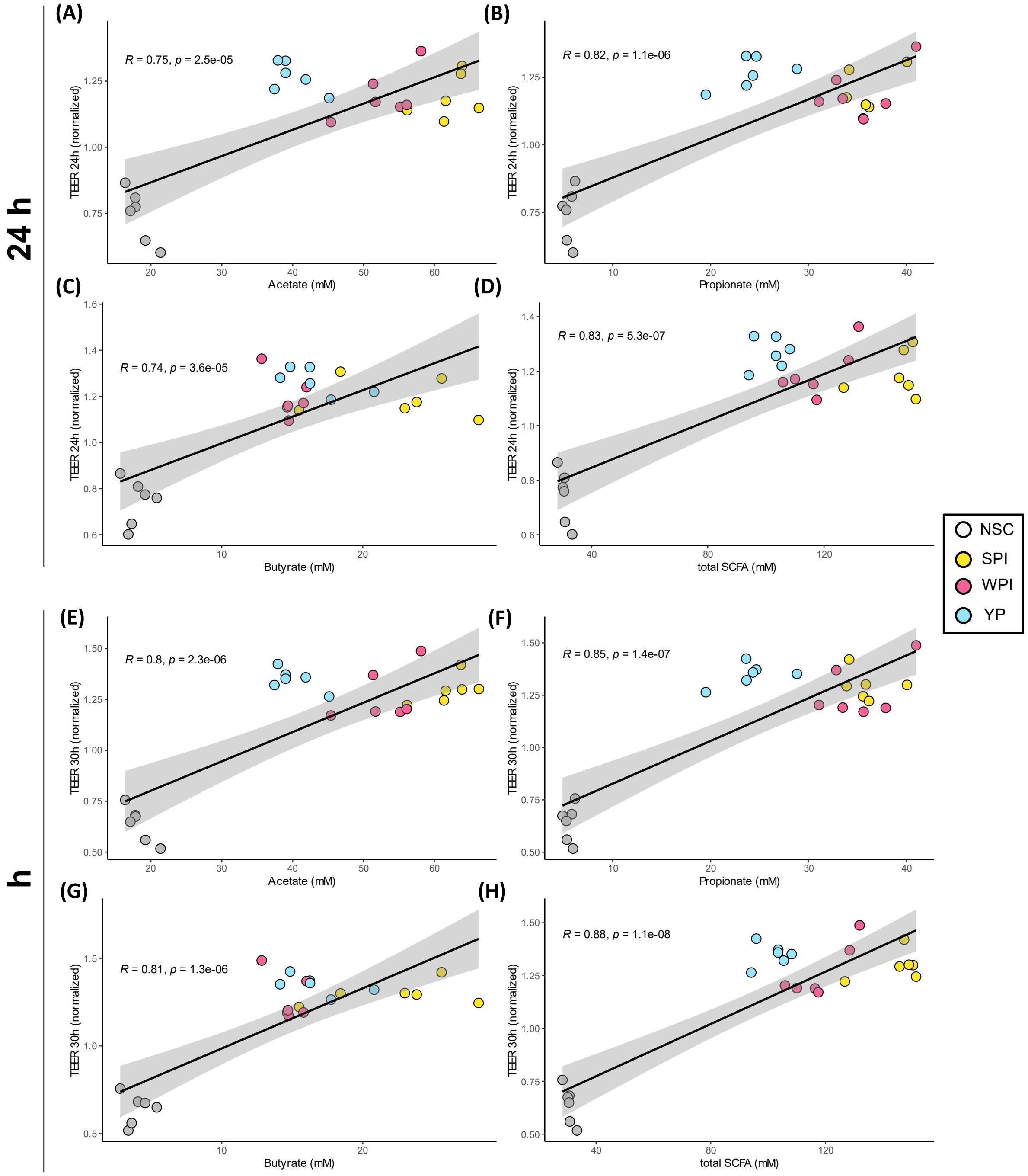

Figure S3. SCFA levels strongly correlated with the improved barrier integrity upon protein treatment (YP, WPI, SBI). Pearson correlation analysis between gut barrier integrity (normalized TEER of the Caco-2 epithelial layer) and SCFA (mM) (**A-D**) under unstressed conditions (24 h) and (**E-H**) stressed conditions (30 h; additional 6 h LPS treatment).

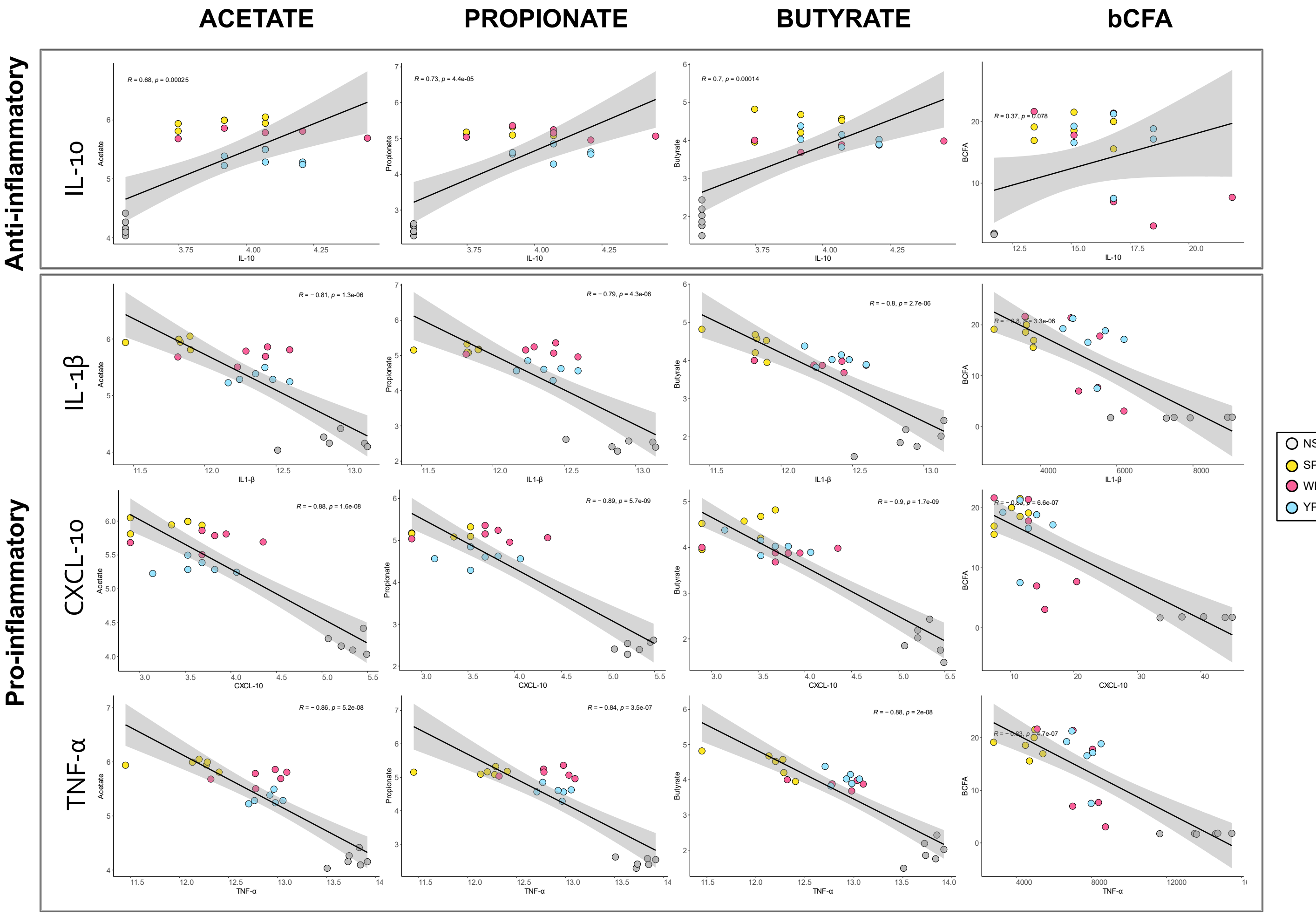

Figure S4. **SCFA levels correlated with the increase of anti-inflammatory and decrease of pro-inflammatory markers upon protein treatment (YP, WPI, SBI).** Pearson correlation analysis between anti-inflammatory (IL-10) or pro-inflammatory markers (TNF-α/IL1-β/CXCL-10) and SCFA/bCFA. The concentration of SCFA and BCFA (mM) and the immune markers (pg/mL) are log_2-transformed.

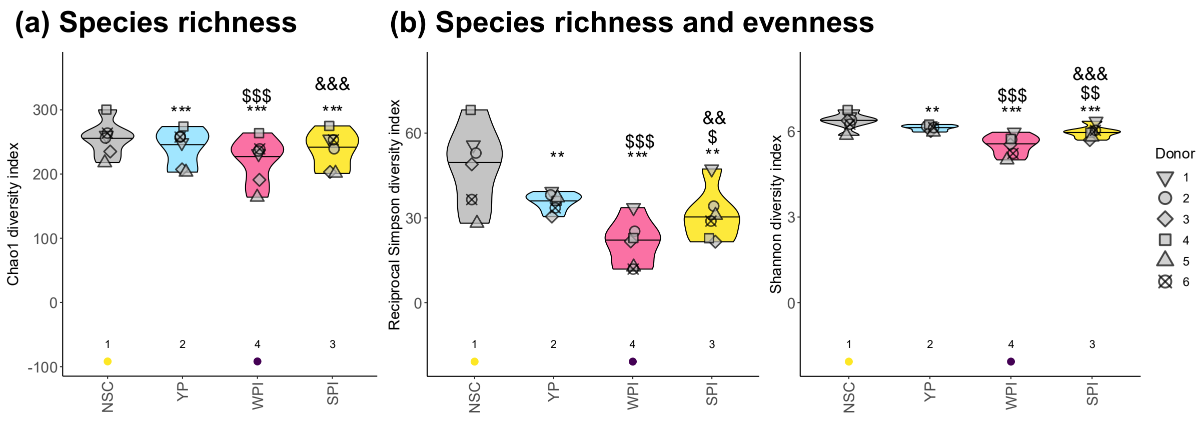

Figure S5. Traditional diversity indices decreased upon protein treatment. The impact on traditional diversity indices for (**a**) richness (Chao1 diversity index) and (**b**) evenness (reciprocal Simpson diversity index/Shannon diversity index). Statistical differences with NSC are indicated with * ($0.10 < p_{adjusted} < 0.20$), ** ($0.05 < p_{adjusted} < 0.10$) or *** ($p_{adjusted} < 0.05$). "$/$$/$$$" indicates differences between YP and WPI or SPI, while "$/$$/$$$" indicates differences between WPI and SPI. Ranks of the average values per treatment are indicated at the bottom, with the lowest average being indicated in purple, and the highest in yellow.

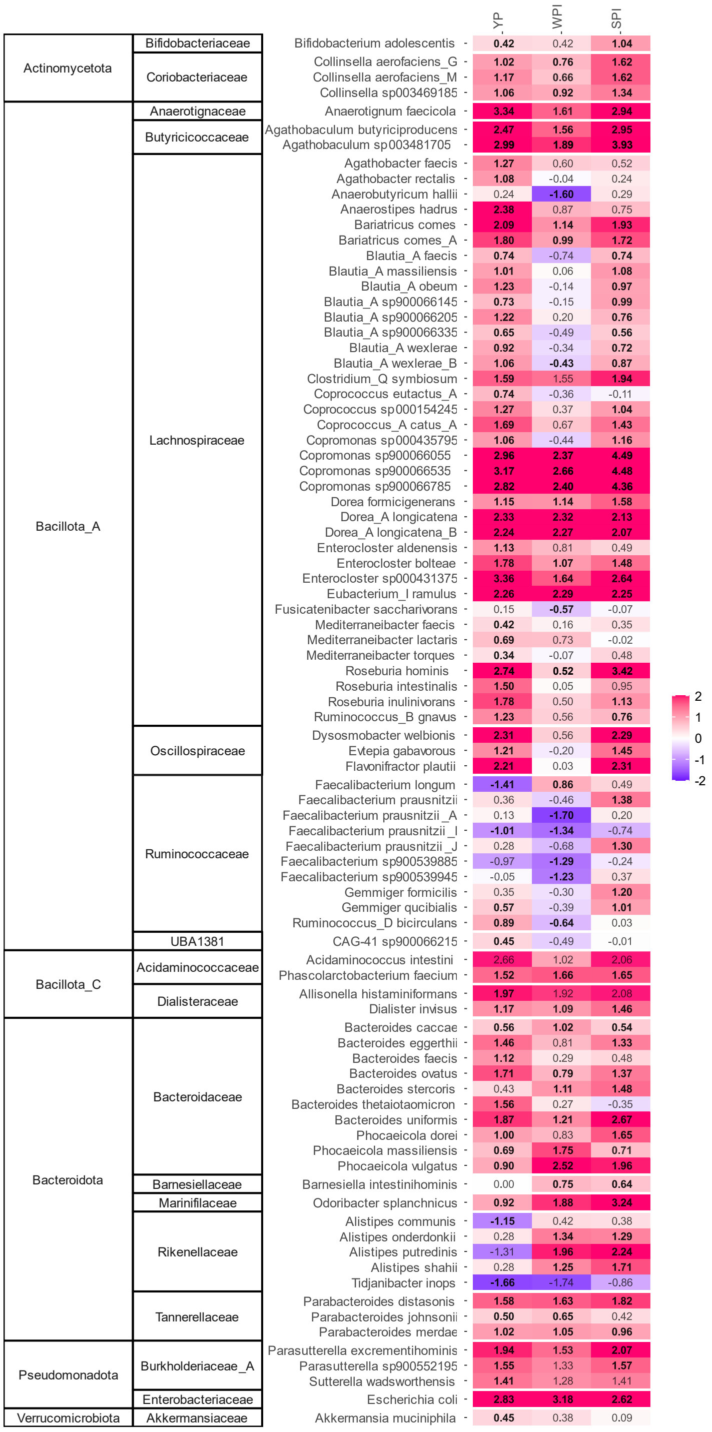

Figure S6. The proteins (YP, WPI, SBI) stimulated specific microbial species in a protein-specific manner. Heatmap based on species that were significantly (FDR = 0.20) affected by any of the treatments, expressed as $\log_2$ (treatment/NSC), averaged over the six test subjects. Values indicated in bold show significant increases (> 0) or decreases (< 0). Corresponding phyla and families are indicated on the left for each species.

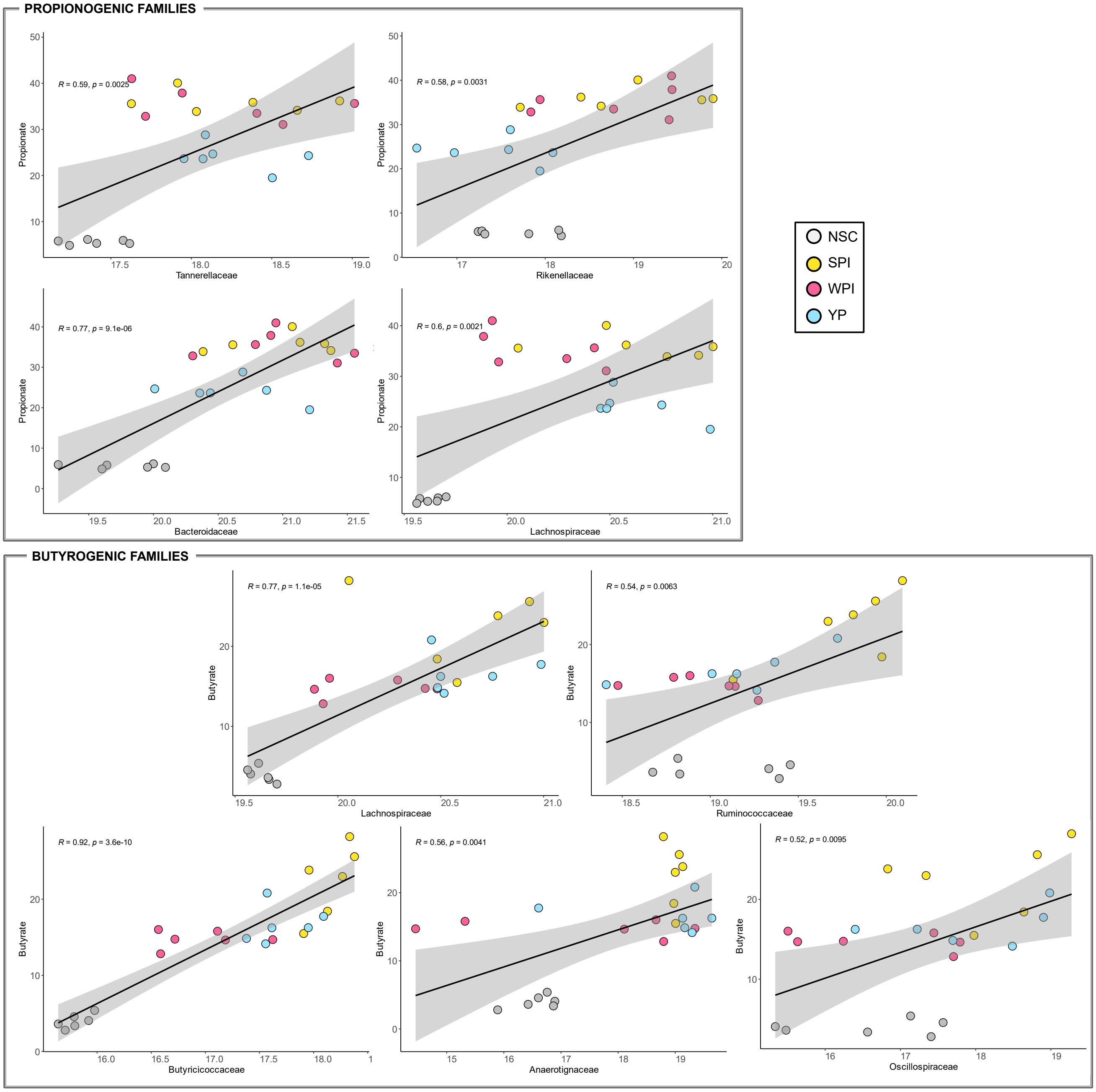

Figure S7. Propionate and butyrate levels correlated with the increased abundances of propionogenic and butyrogenic families upon protein treatment (YP, WPI, SBI). Pearson correlation analysis between the log_2-transformed abundances of different families and propionate and butyrate (mM).

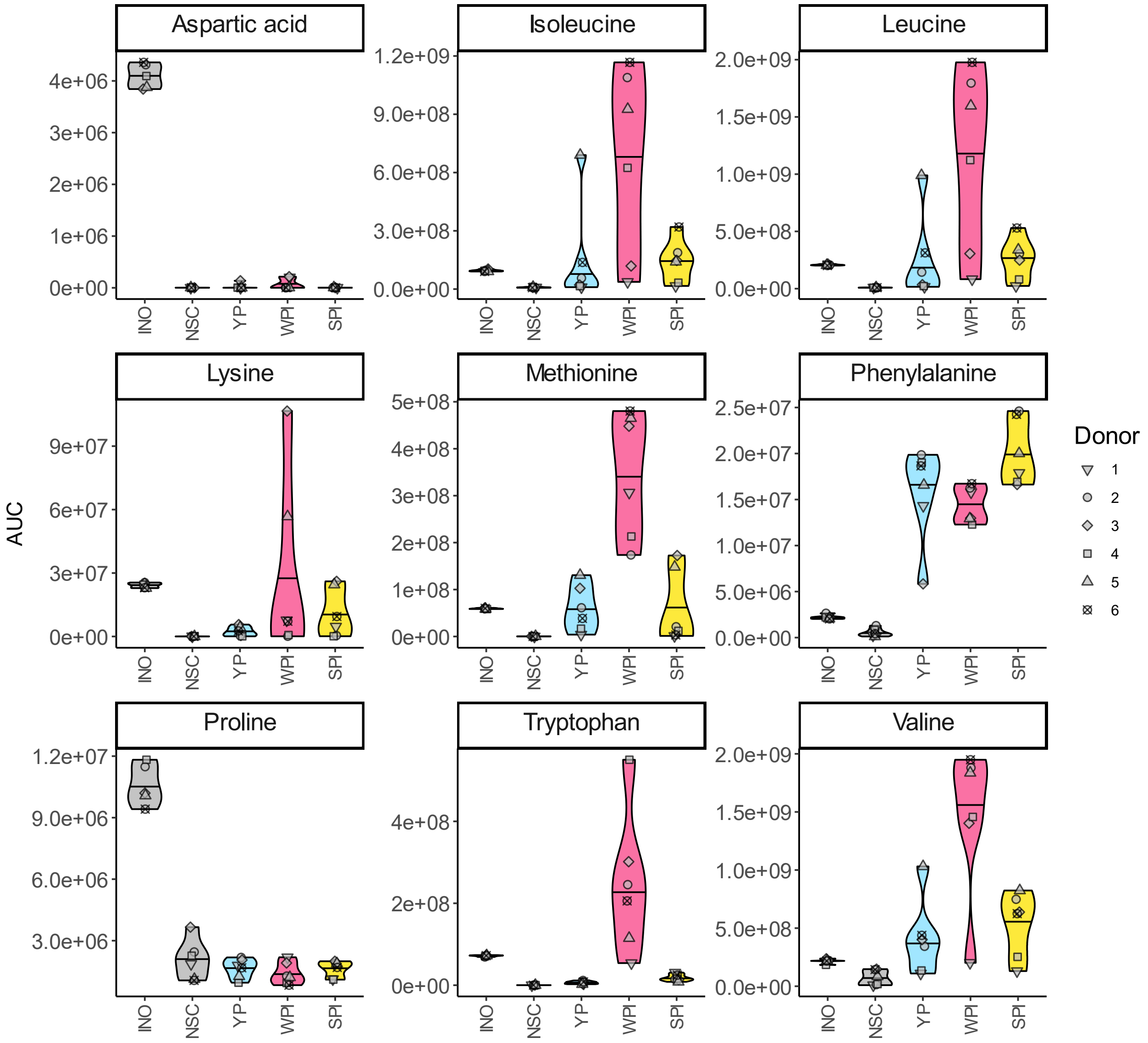

Figure S8. While amino acids were efficiently consumed under control conditions (INO → NSC), the proteins (YP, WPI, SBI) increased some amino acids in a protein-specific manner. Level 1/2a-annotated amino acids detected in the untargeted LC-MS/MS analysis, both at 0h (INO) and upon 24 h of colonic incubation. AUC: area under the curve.

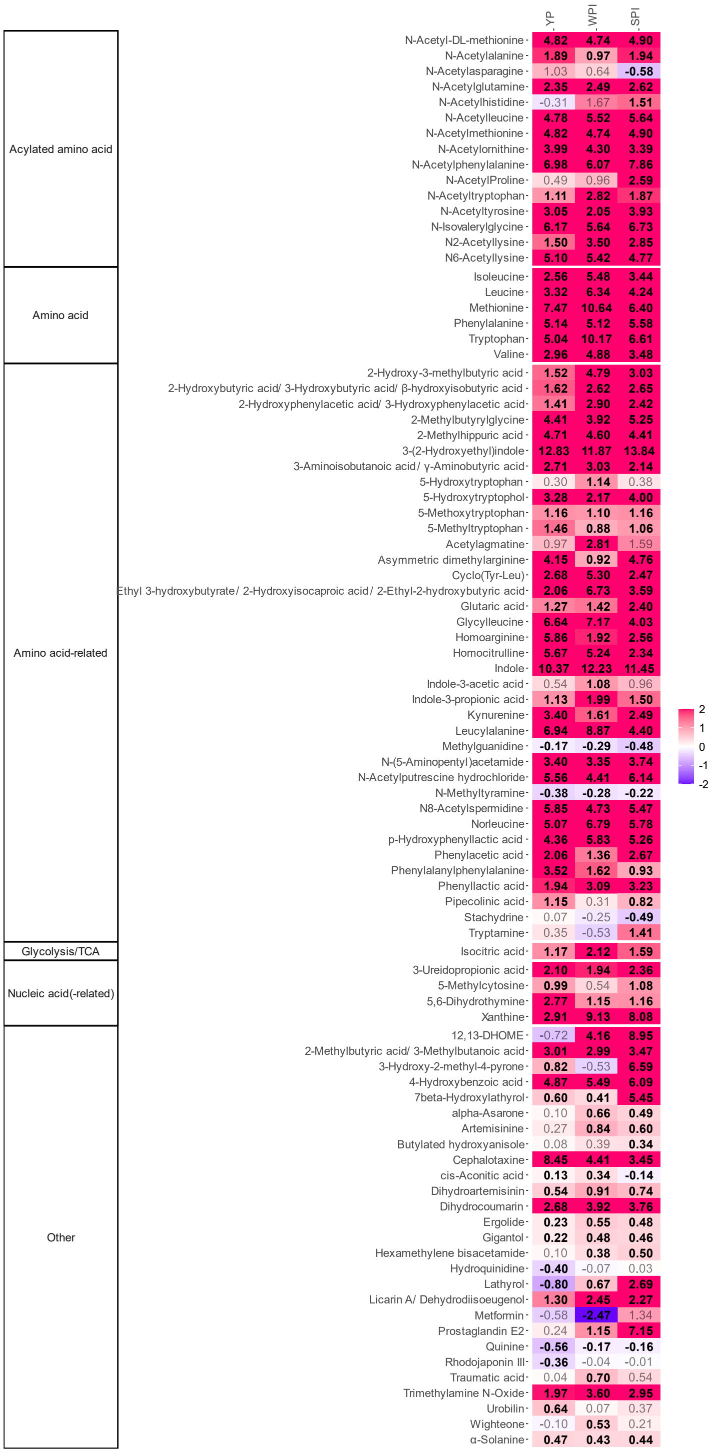

Figure S9. The proteins (YP, WPI, SBI) stimulated specific microbial metabolites in a protein-specific manner. Heatmap based on level 1/2a-annotated metabolites that were produced during the 24 h incubation and significantly (FDR = 0.20) affected by any of the treatments, expressed as $\log_2$ (treatment/NSC), averaged over the six test subjects. Values indicated in bold show significant increases (> 0) or decreases (< 0).

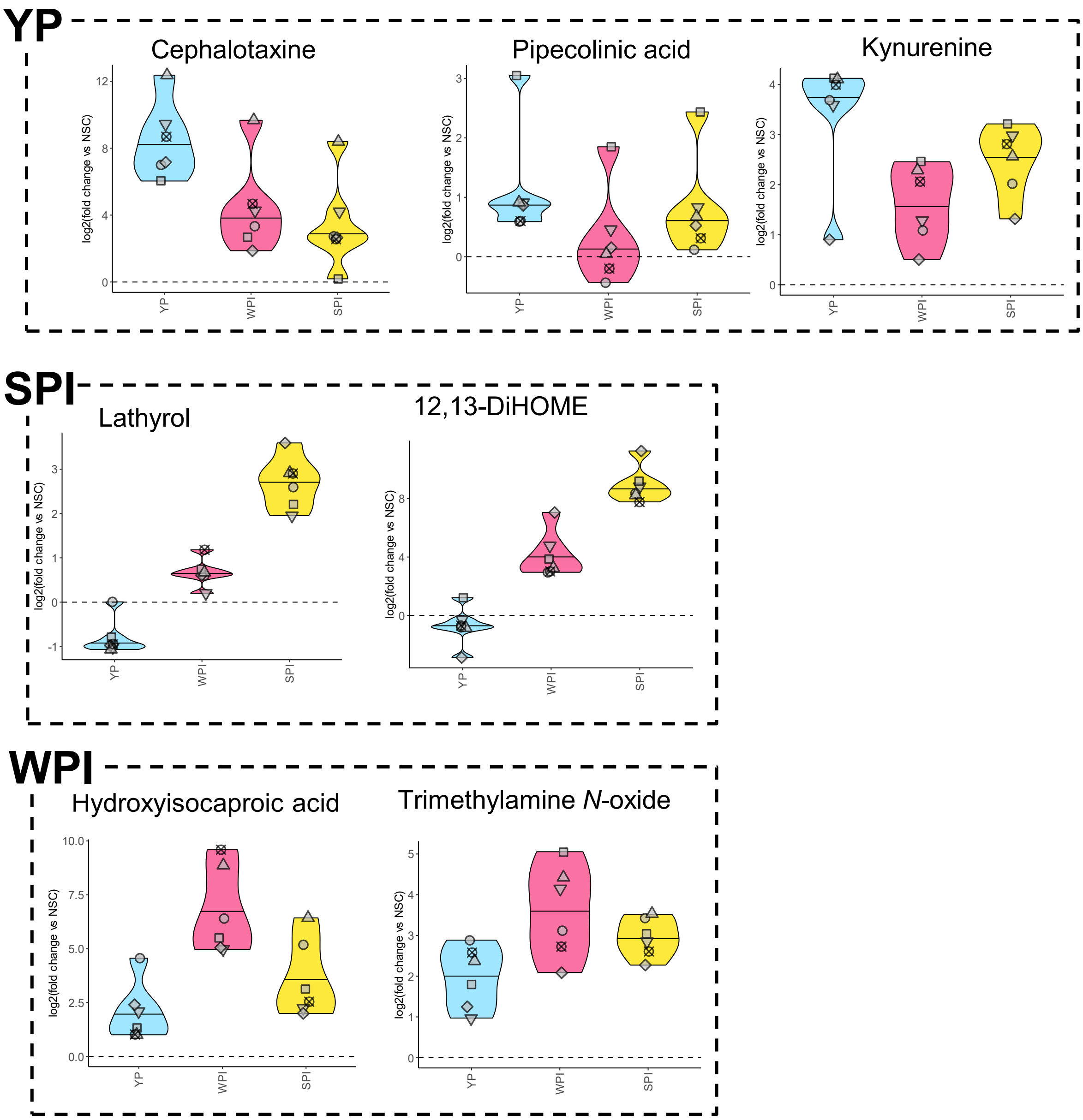

Figure S10. The proteins (YP, WPI, SPI) stimulated additional metabolites in a protein-specific manner. A selection of additional, notable metabolites stimulated in a protein-specific manner is presented in dedicated violin plots.

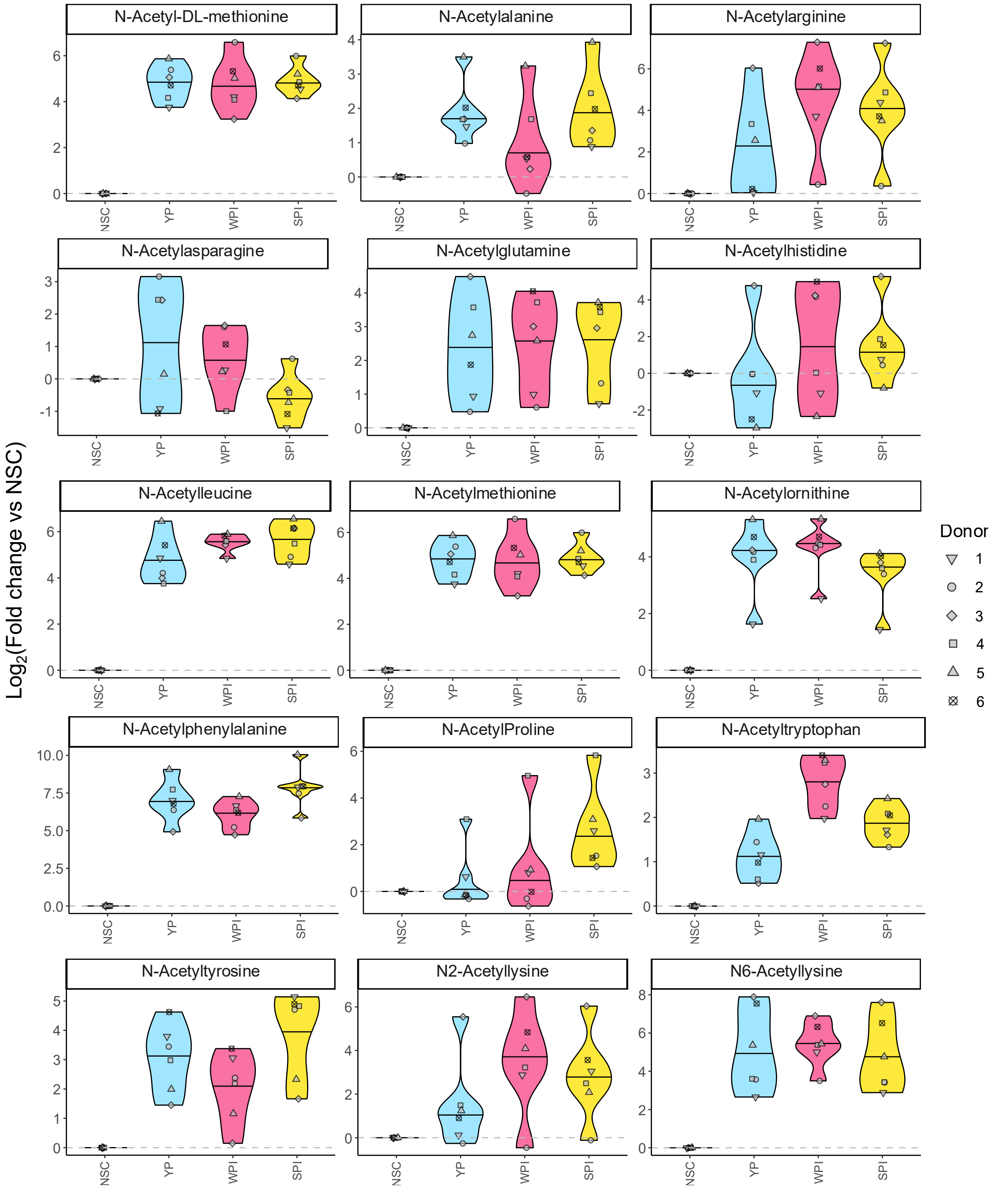

Figure S11. The proteins (YP, WPI, SPI) stimulated *N*-acetylated amino acids in a protein-specific manner. Levels of *N*-acetylated amino acids that (i) increased by any of the proteins at 24 h compared to the NSC at 0h (suggesting production by gut microbes and/or presence in any of the proteins) and (ii) were significantly different for protein treatments compared to NSC at 24 h (FDR = 0.20), expressed as $\log_2$ (treatment/NSC), for each of the six 50-65y male adults.

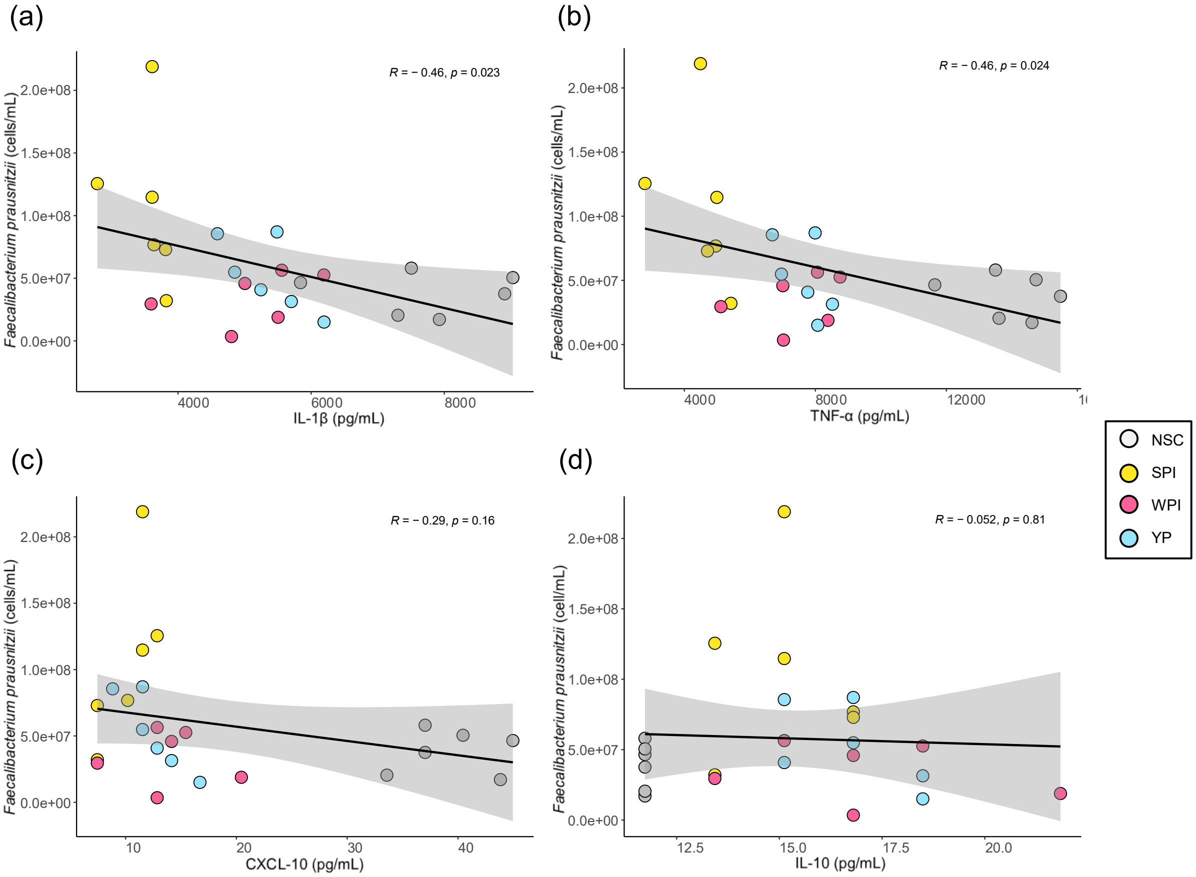

Figure S12. *Faecalibacterium prausnitzii* abundance was inversely correlated with the pro-inflammatory markers IL-1β and TNF-α upon protein treatment (YP, WPI, SBI). Correlation analysis based on Pearson correlation coefficient between *Faecalibacterium prausnitzii* abundances with the pro-inflammatory markers (**a**) IL-1β, (**b**) TNF-α, (**c**) CXCL-10 and the anti-inflammatory marker (**d**) IL-10 across four different study arms and six different donors.
